# Supplementary material for: Novel Derivatives of Eugenol as a New Class of PPARγ Agonists in Treating Inflammation: Design, Synthesis, SAR Analysis and In Vitro Anti-Inflammatory Activity
Source: Molecules. 2023 May 5;28(9):3899. doi: 10.3390/molecules28093899 (PMC10180488; doi:10.3390/molecules28093899)
Supplement: Supplementary file 1 [file molecules-28-03899-s001.zip › molecules-2248337-supplementary.pdf]

Novel Derivatives of Eugenol as a New Class of PPARγ Agonists in Treating Inflammation: Design, Synthesis, SAR Analysis and In Vitro Anti-Inflammatory Activity

Noor Fathima Anjum [1,2], Dhivya Shanmugarajan [2], B. R. Prashantha Kumar [2], Syed Faizan [2], Priya Durai [2], Ruby Mariam Raju [2], Saleem Javid [1,2] and Madhusudan N. Purohit [2,*]

[1]*Department of Pharmaceutical Chemistry, Farooqia College of Pharmacy, Mysuru 570 015, India*

[2]*Department of Pharmaceutical Chemistry, JSS College of Pharmacy, JSS Academy of Higher Education &Research, Mysuru 570 015, India*

Supplementary Material

CONTENTS **Page No.**

1. **Spectra... 2-13**
2. **TR-FRET Assay.. 14-16**

Spectra of synthesized compounds

Figure S1. IR, ^{H}NMR, ^{13}CNMR & Mass Spectra of compound 1a

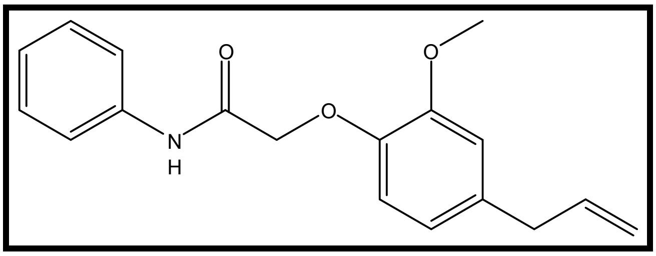

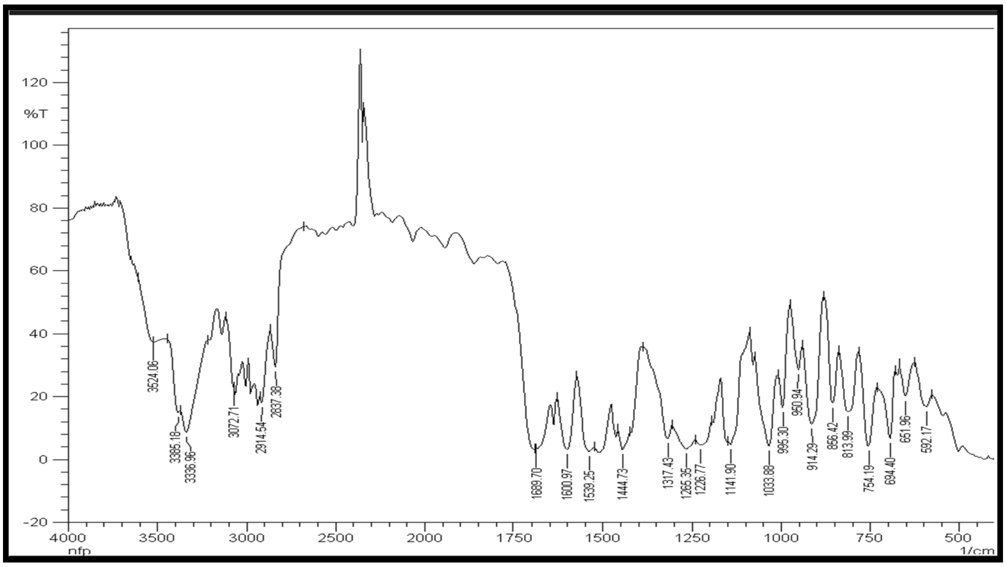

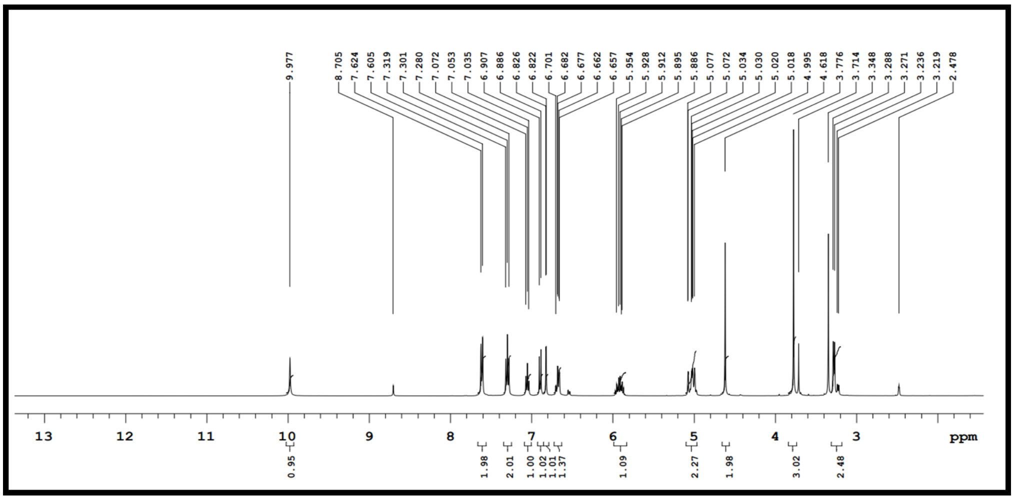

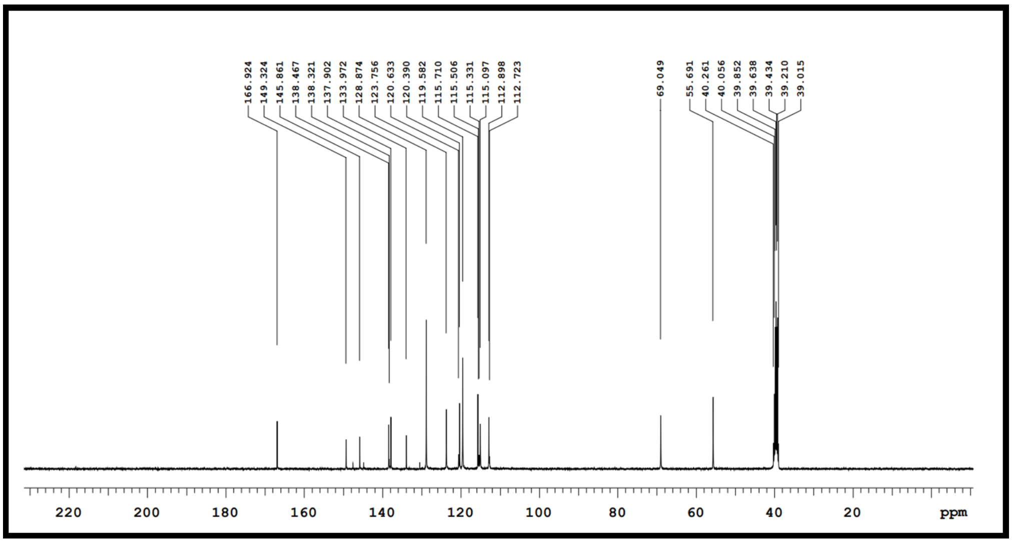

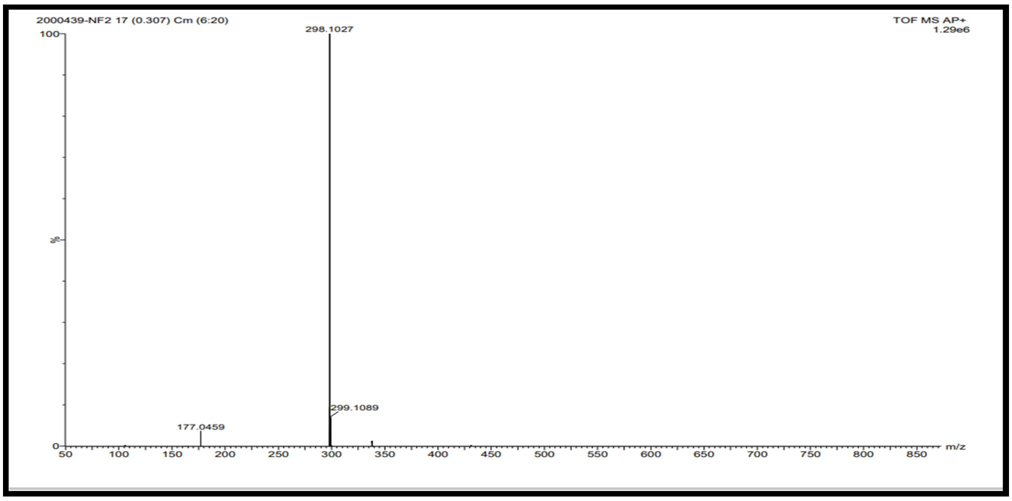

Figure S2. IR, ^{H}NMR, ^{13}CNMR & Mass Spectra of compound 1b

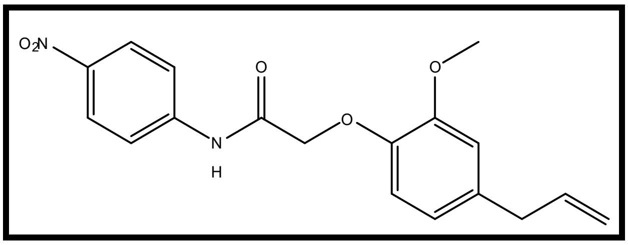

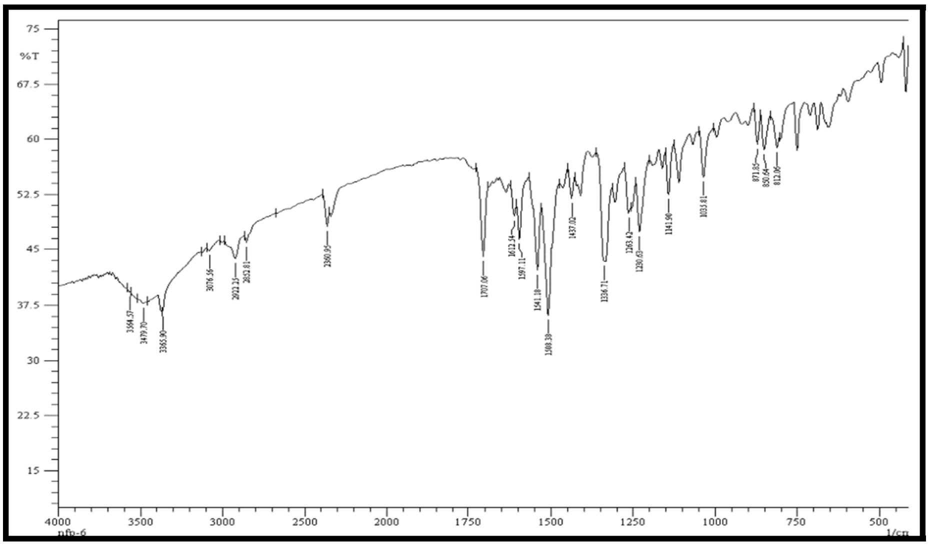
75
%T
67.5
60
52.5
45
37.5
30
22.5
15
3564.57
3479.70
3365.90
3076.56
2922.25
2852.81
2360.95
1707.06
1612.54
1597.11
1541.18
1508.38
1437.02
1336.71
1263.42
1230.63
1141.90
1035.81
871.85
850.64
812.06
4000
3500
3000
2500
2000
1750
1500
1250
1000
750
500
nfb-6
1/cm

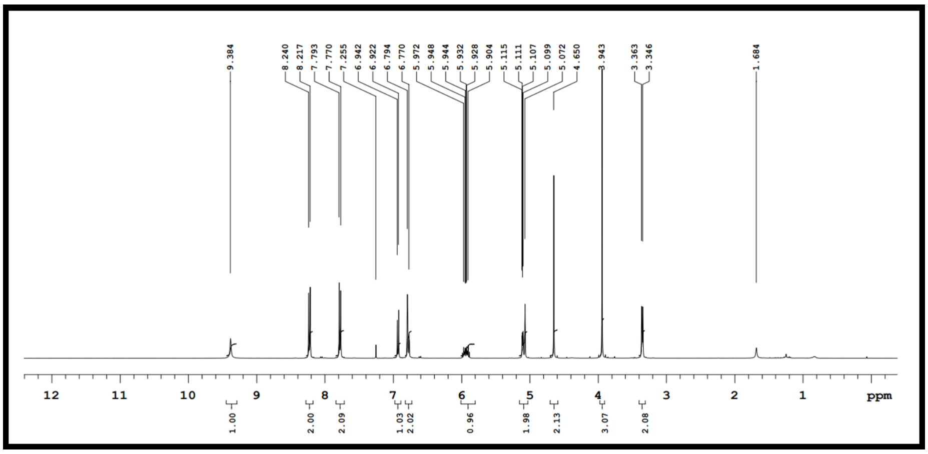
9.384
8.240
8.217
7.793
7.770
7.255
6.942
6.922
6.794
6.770
5.972
5.948
5.944
5.932
5.928
5.904
5.115
5.111
5.107
5.099
5.072
4.650
3.943
3.363
3.346
1.684
12
11
10
9
8
7
6
5
4
3
2
1
ppm
1.00
2.00
2.09
1.03
2.02
0.96
1.98
2.13
3.07
2.08

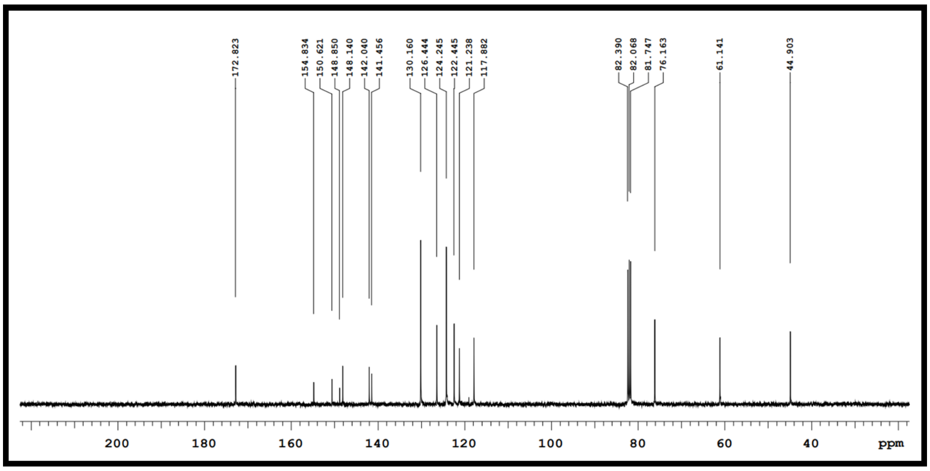
172.823
154.834
150.621
148.850
148.140
142.040
141.456
130.160
126.444
124.245
122.445
121.238
117.882
82.390
82.068
81.747
76.163
61.141
44.903
200
180
160
140
120
100
80
60
40
ppm

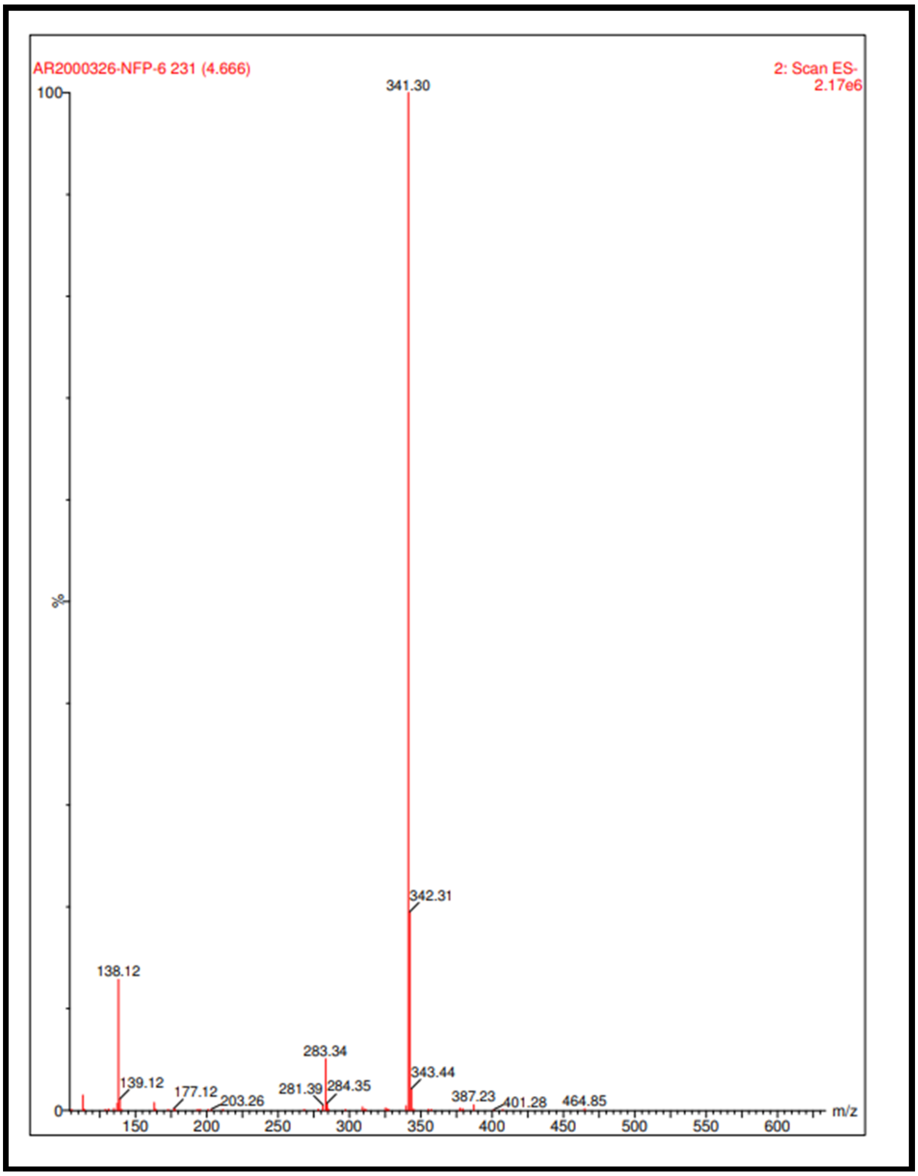

Figure S3. IR, HNMR, 13CNMR & Mass Spectra of compound 1c

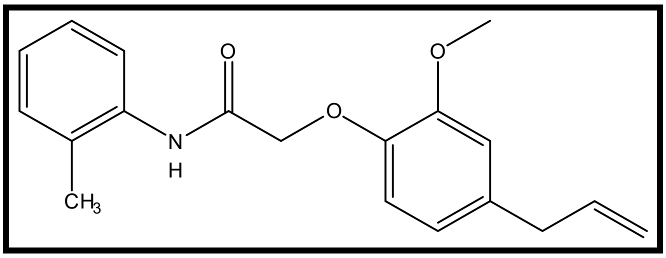

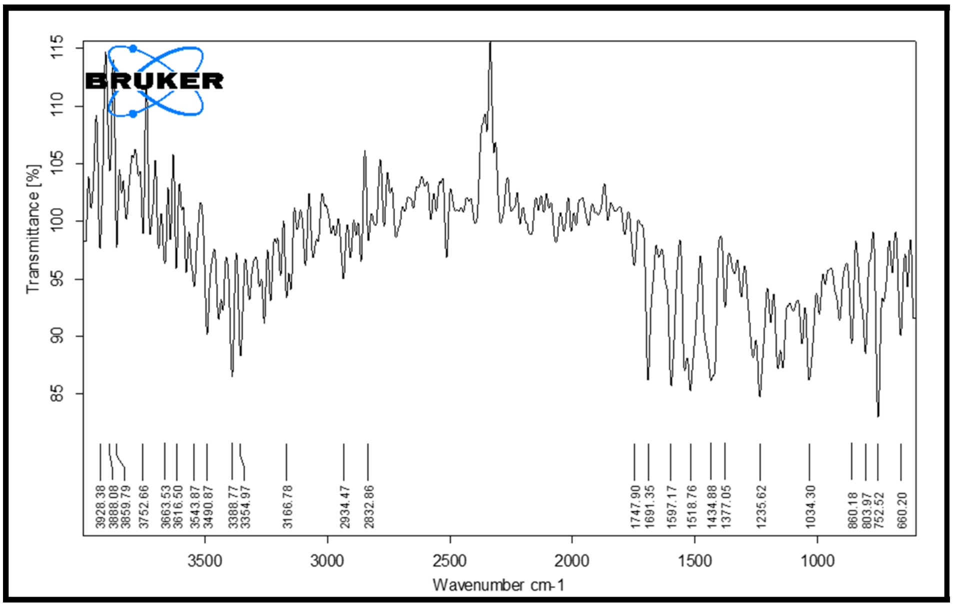

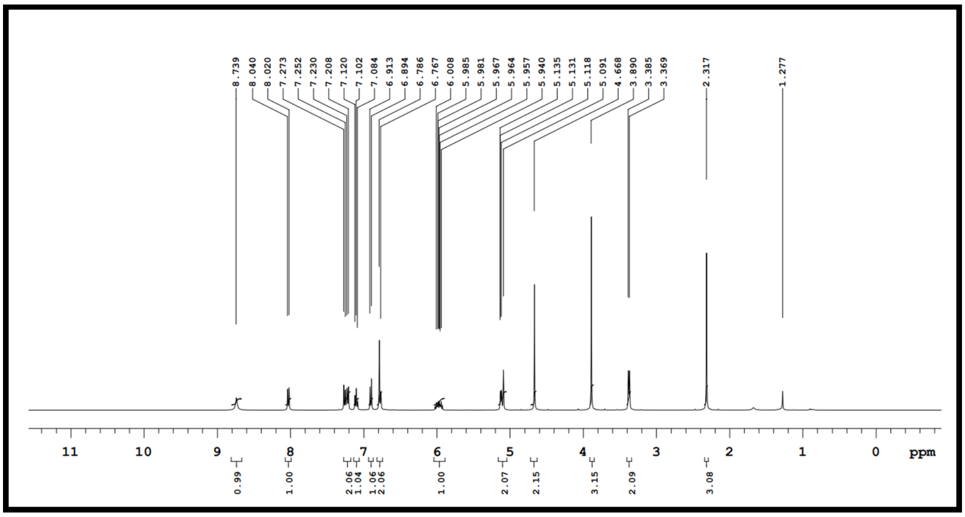

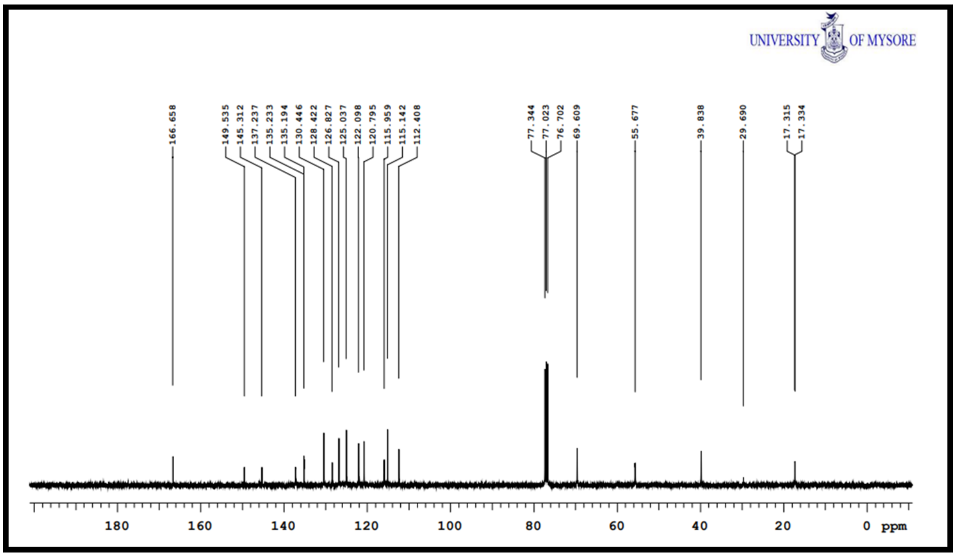

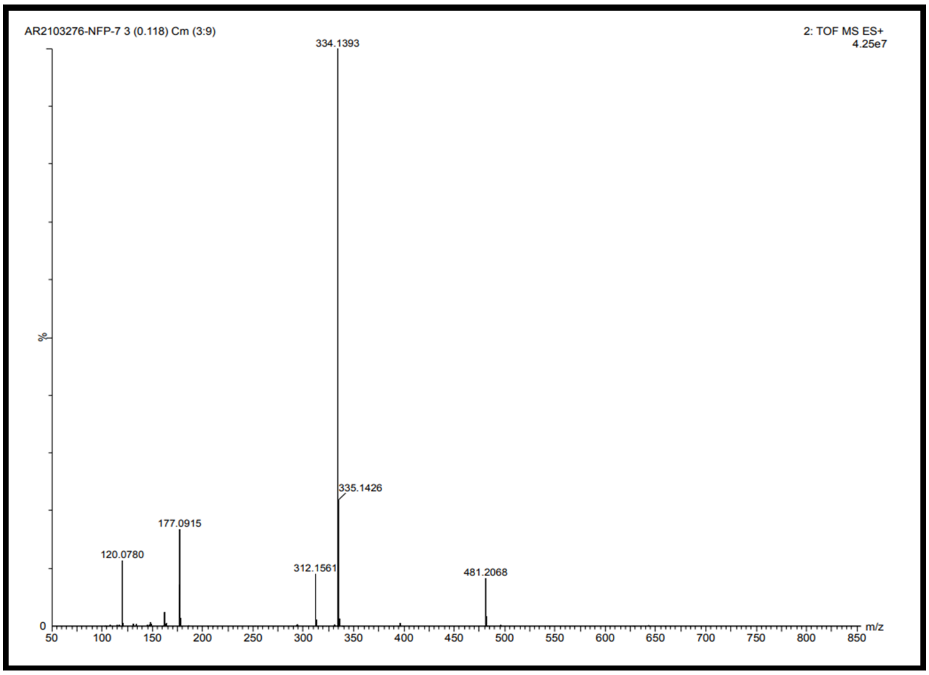

Figure S4. IR, HNMR, 13CNMR & Mass Spectra of compound 1d

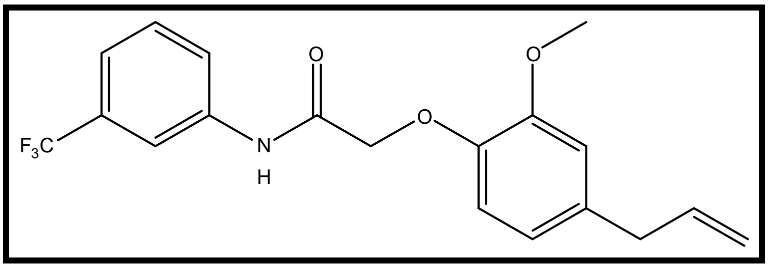

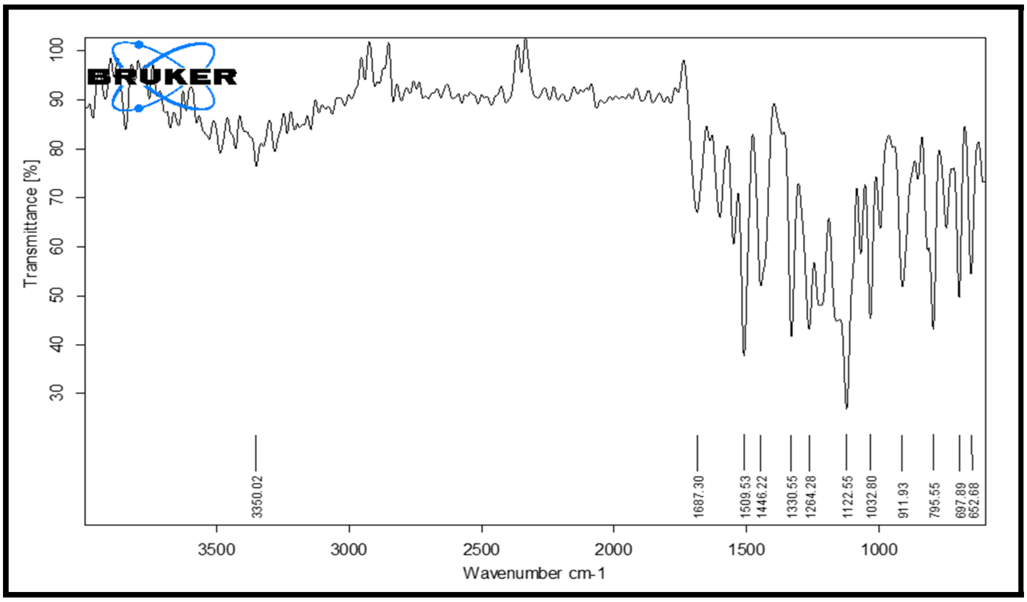

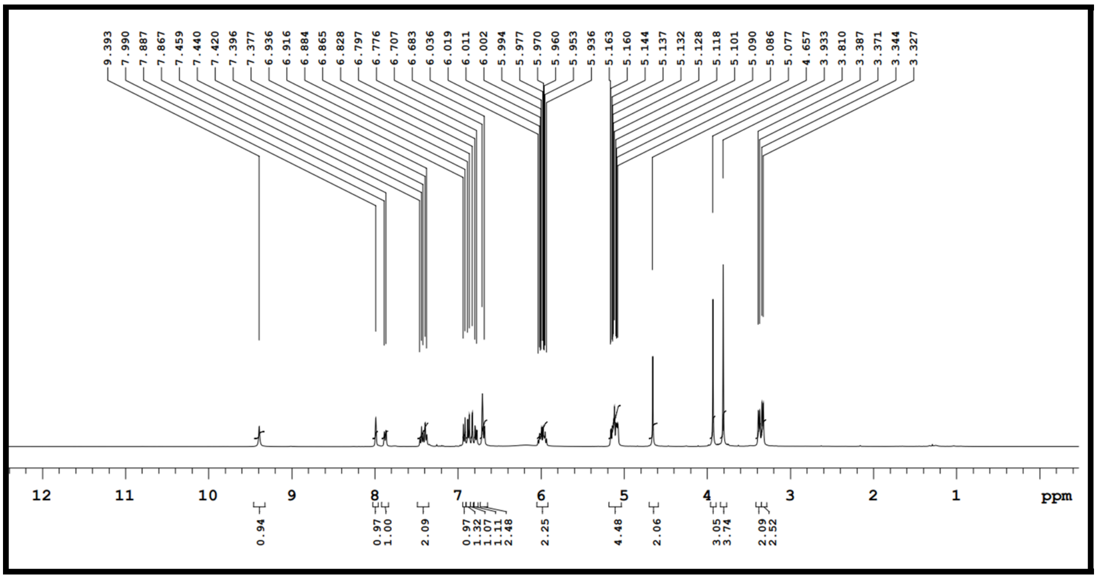

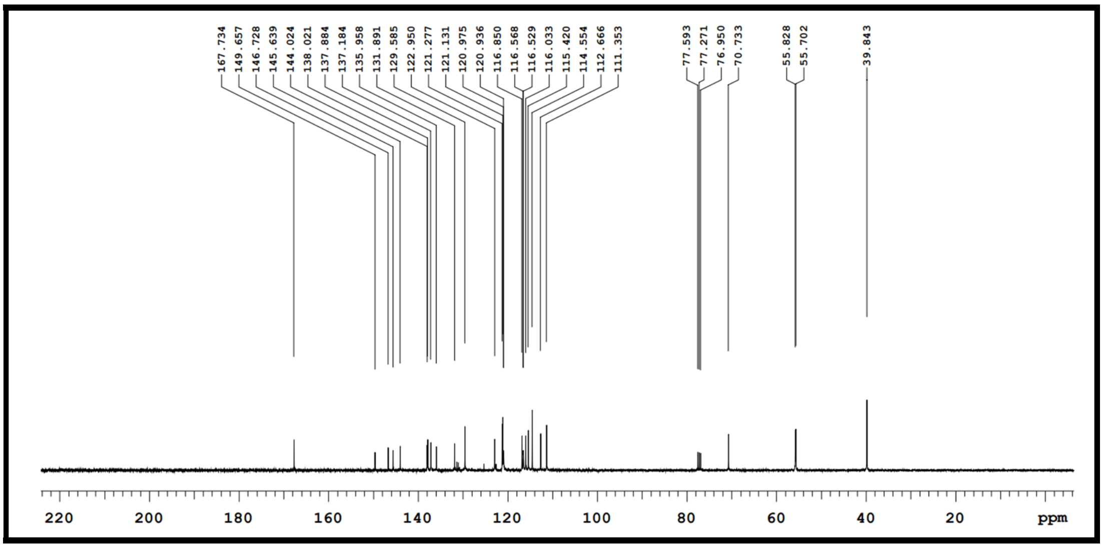

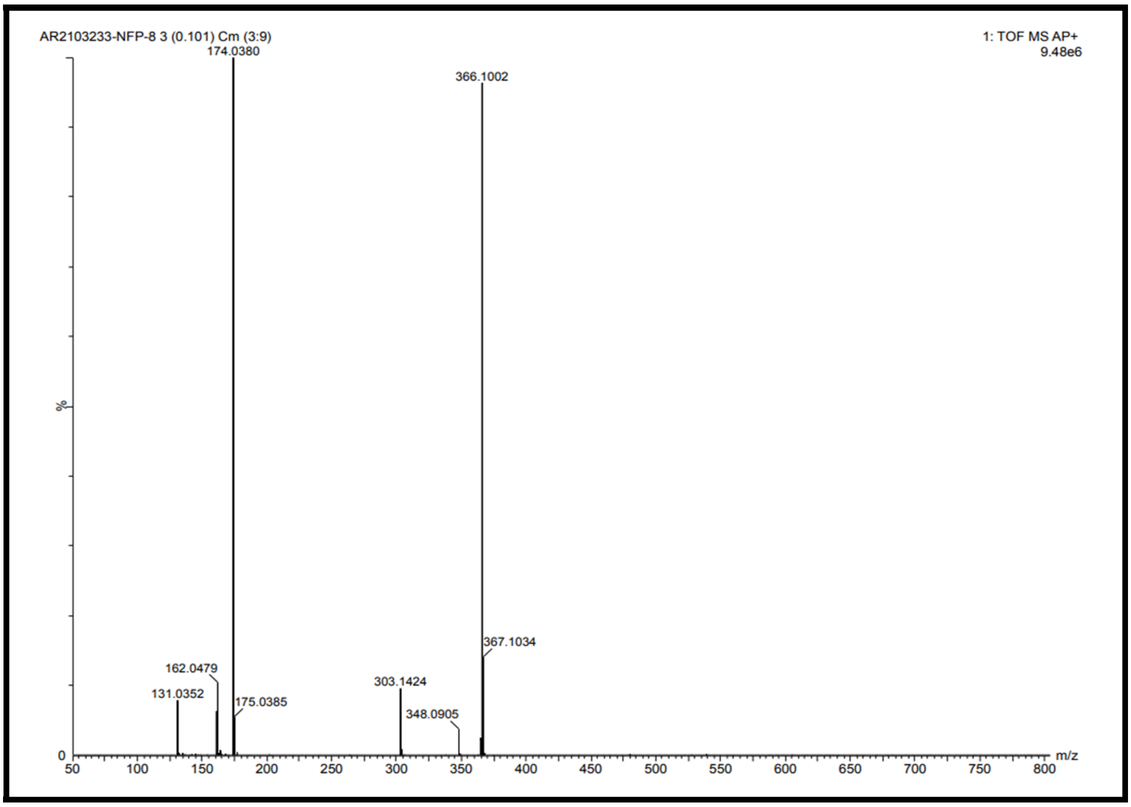

Figure S5. IR, HNMR, 13CNMR & Mass Spectra of compound 1e

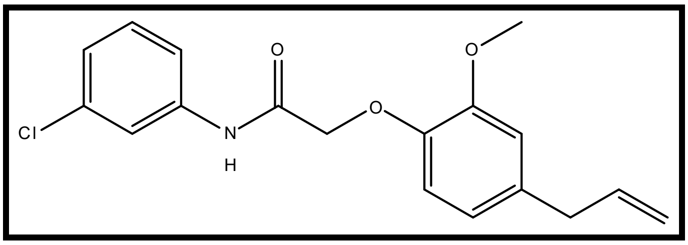

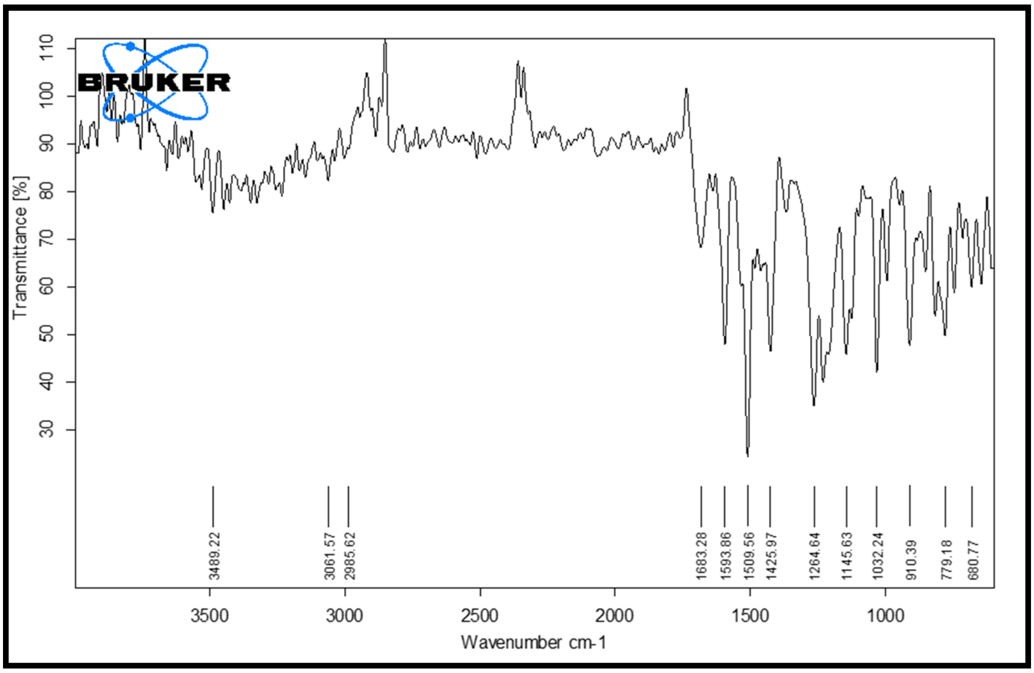
Transmittance [%]
Wavenumber cm-1
3489.22
3061.57
2985.62
1683.28
1593.86
1509.56
1425.97
1264.64
1145.63
1032.24
910.39
779.18
680.77

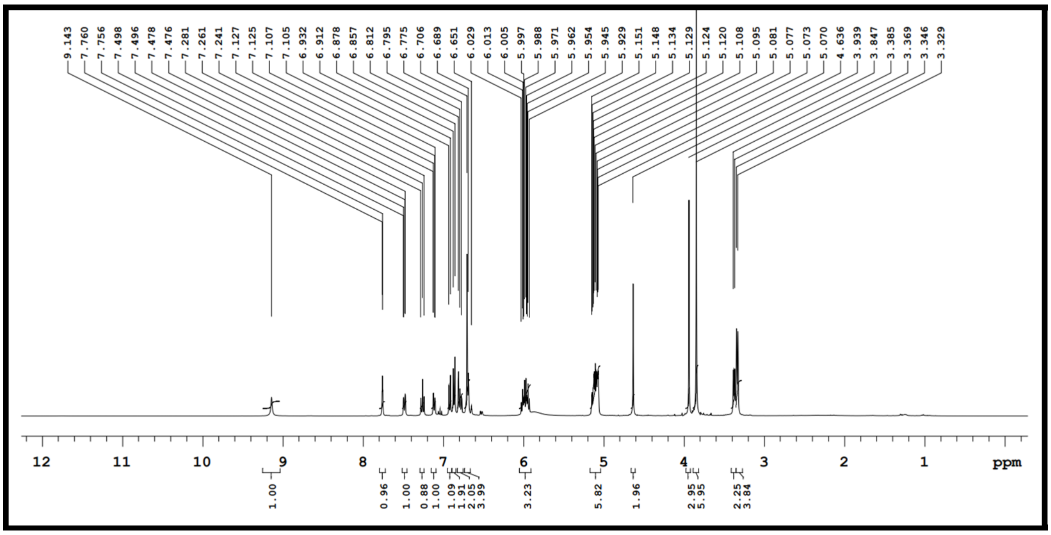
9.143 7.760 7.756 7.498 7.496 7.478 7.476 7.281 7.261 7.241 7.127 7.125 7.107 7.105 6.932 6.912 6.878 6.857 6.812 6.795 6.775 6.706 6.689 6.651 6.029 6.013 6.005 5.997 5.988 5.971 5.962 5.954 5.945 5.929 5.151 5.148 5.134 5.129 5.124 5.120 5.108 5.095 5.081 5.077 5.073 5.070 4.636 3.939 3.847 3.385 3.369 3.346 3.329
ppm
1.00 0.96 1.00 0.88 1.00 1.09 1.91 2.05 3.99 3.23 5.82 1.96 2.95 5.95 2.25 3.84

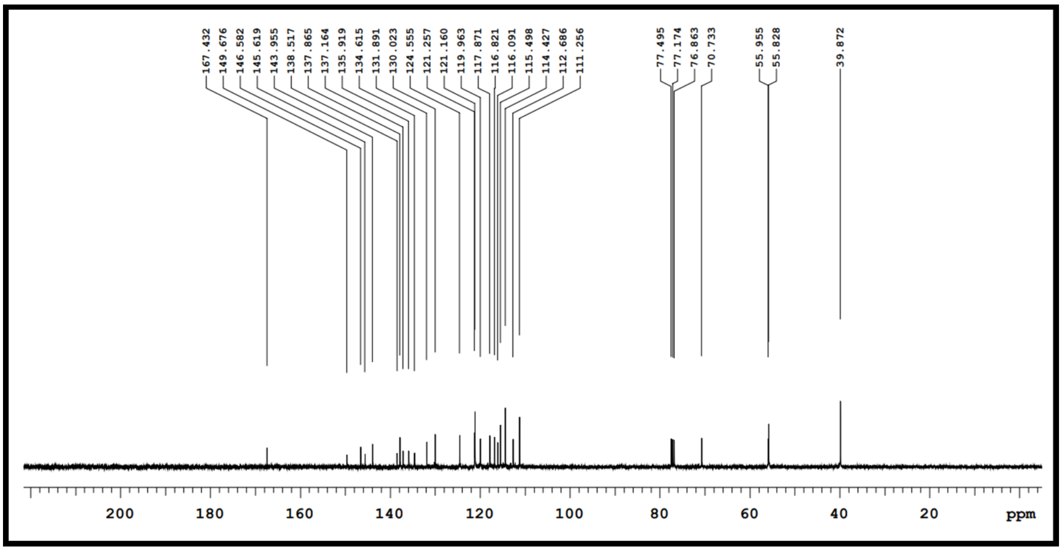
167.432 149.676 146.582 145.619 143.955 138.517 137.865 137.164 135.919 134.615 131.891 130.023 124.555 121.257 121.160 119.963 117.871 116.821 116.091 115.498 114.427 112.686 111.256 77.495 77.174 76.863 70.733 55.955 55.828 39.872
ppm

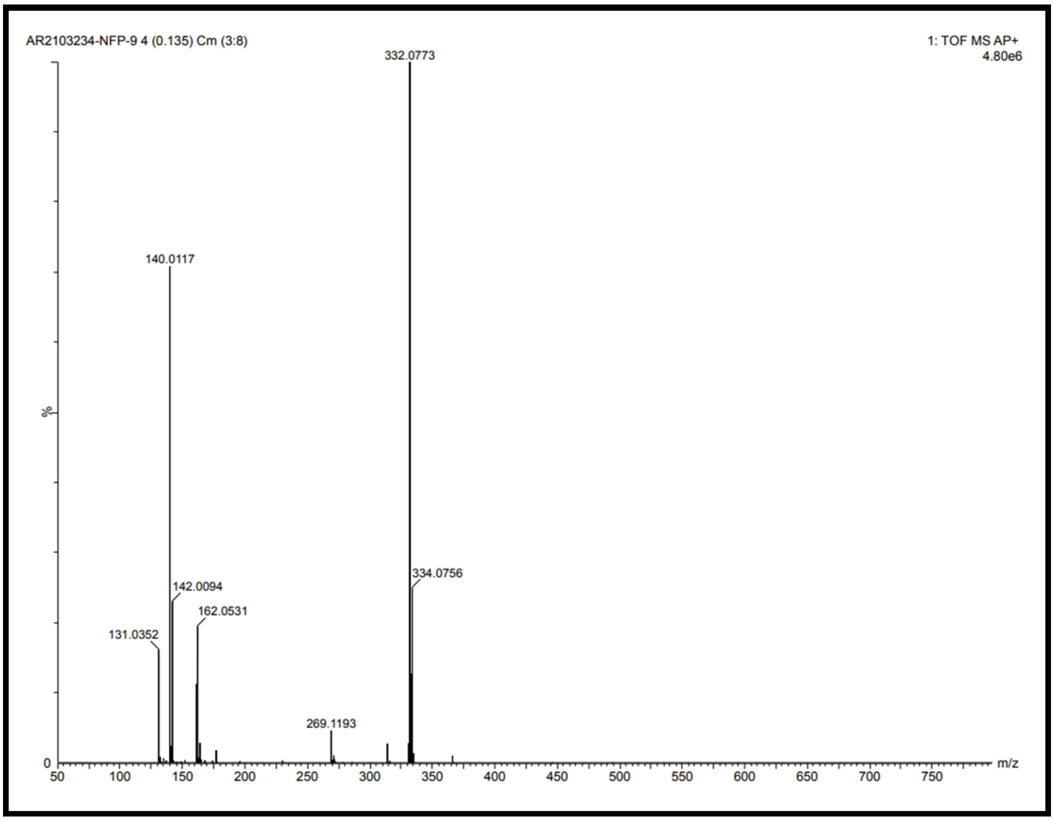

Figure S6. IR, HNMR, 13CNMR & Mass Spectra of compound 1f

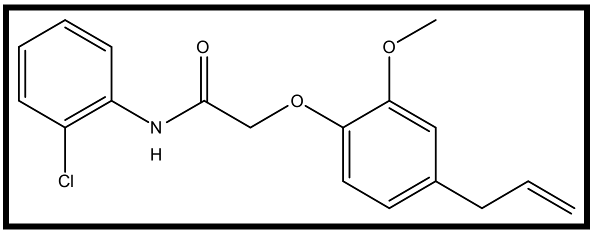

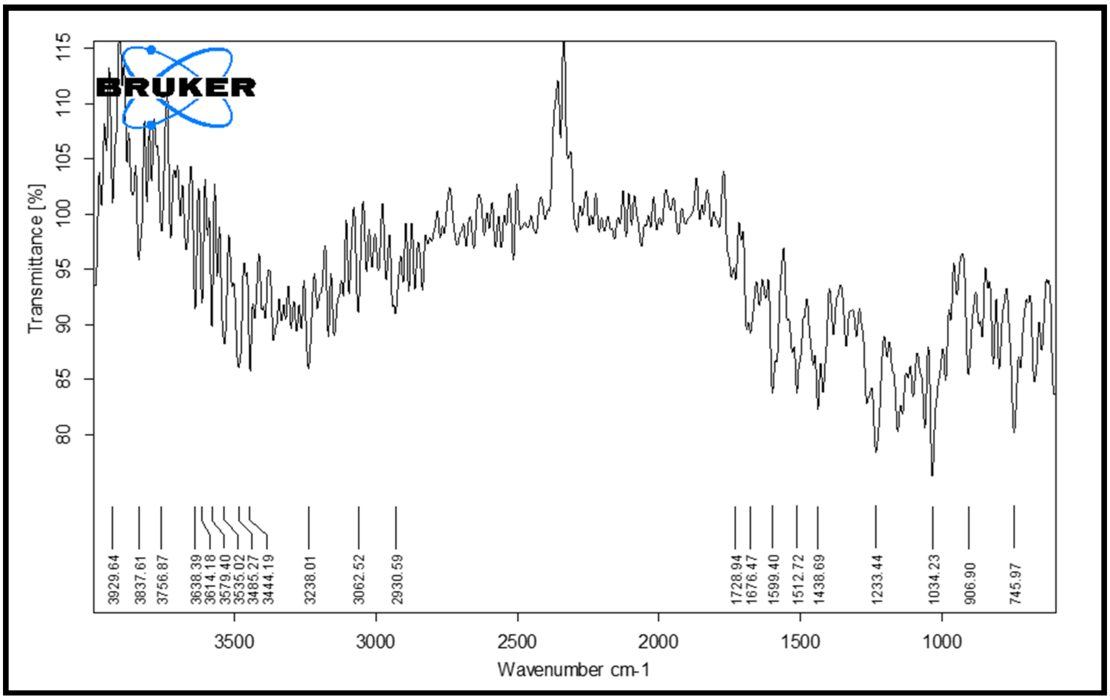
BRUKER
Transmittance [%]
115
110
105
100
95
90
85
80
3929.64
3837.61
3756.87
3638.39
3614.18
3579.40
3535.02
3485.27
3444.19
3238.01
3062.52
2930.59
1728.94
1676.47
1599.40
1512.72
1438.69
1233.44
1034.23
906.90
745.97
3500
3000
2500
2000
1500
1000
Wavenumber cm-1

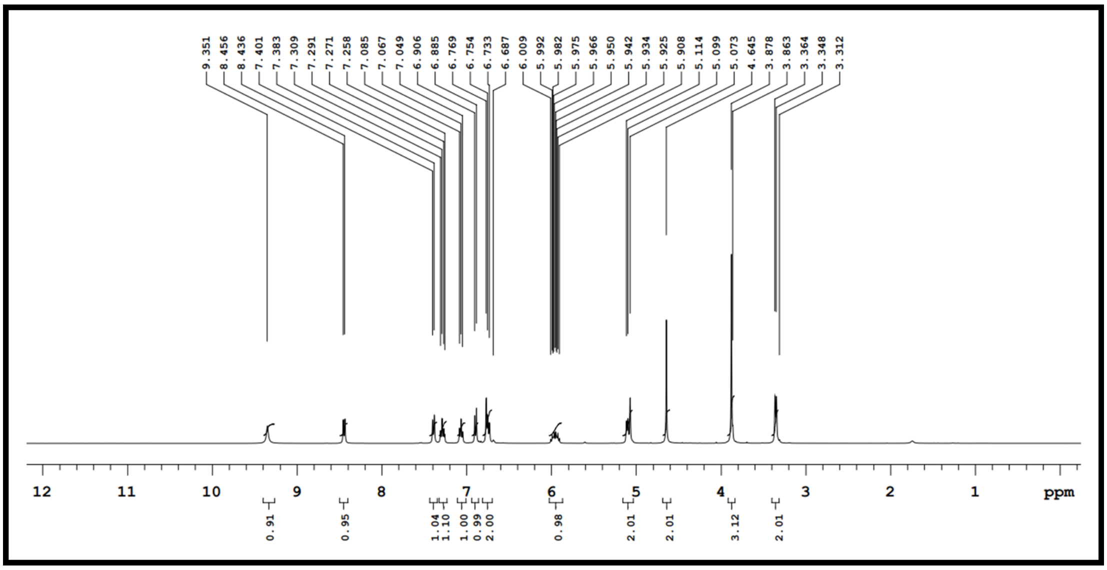
9.351
8.456
8.436
7.401
7.383
7.309
7.291
7.271
7.258
7.085
7.067
7.049
6.906
6.885
6.769
6.754
6.733
6.687
6.009
5.992
5.982
5.975
5.966
5.950
5.942
5.934
5.925
5.908
5.114
5.099
5.073
4.645
3.878
3.863
3.364
3.348
3.312
12
11
10
9
8
7
6
5
4
3
2
1
ppm
0.91
0.95
1.04
1.10
1.00
0.99
2.00
0.98
2.01
2.01
3.12
2.01

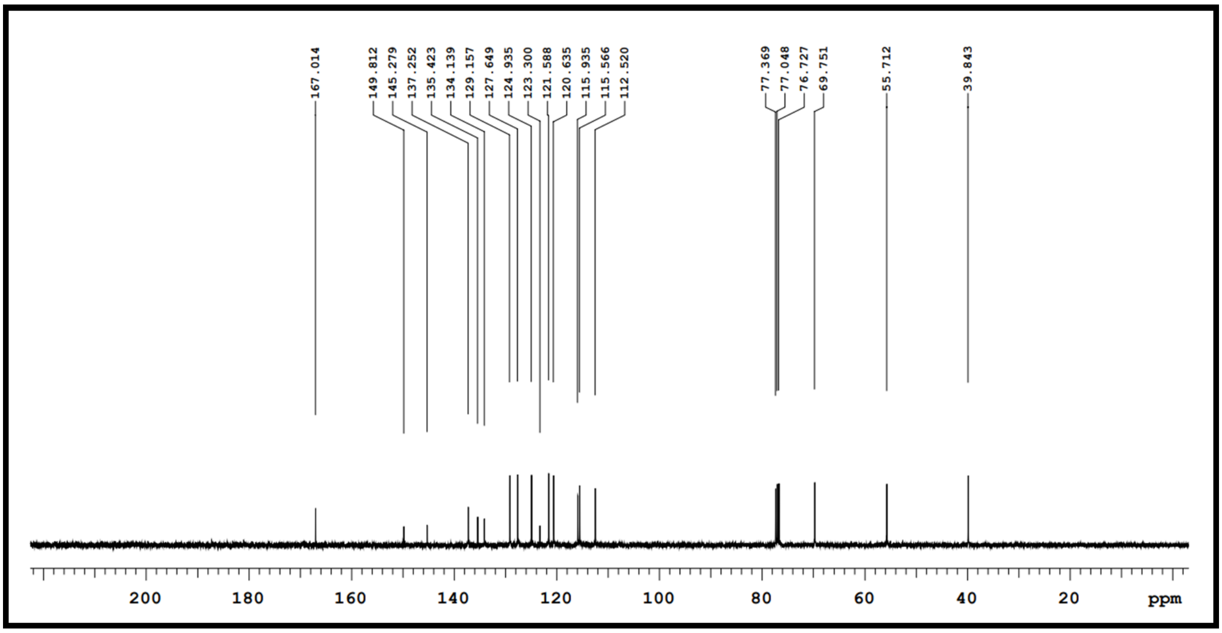

167.014
149.812
145.279
137.252
135.423
134.139
129.157
127.649
124.935
123.300
121.588
120.635
115.935
115.566
112.520
77.369
77.048
76.727
69.751
55.712
39.843
200
180
160
140
120
100
80
60
40
20
ppm

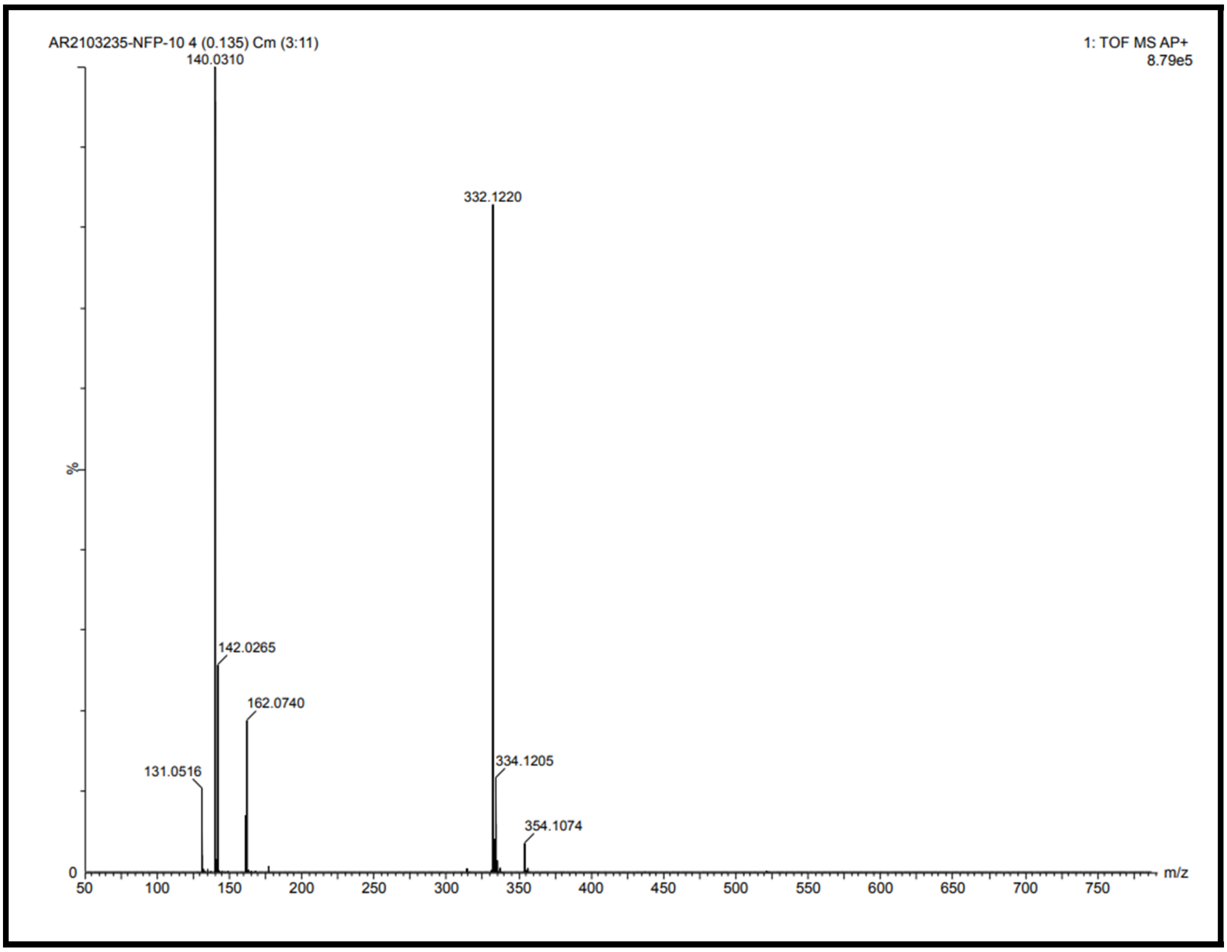

AR2103235-NFP-10 4 (0.135) Cm (3:11)
1: TOF MS AP+
8.79e5
140.0310
332.1220
142.0265
162.0740
131.0516
334.1205
354.1074
%
0
m/z
50
100
150
200
250
300
350
400
450
500
550
600
650
700
750

TR-FRET Assay:

Compound 1a

SL No.	1a (Concentration (μM)	Raw Data (RLU)	Raw Data - Background (RLU)
1	100	0.245	0.01
2	20	1.047	0.813
3	4	1.717	1.483
4	0.8	1.899	1.664
5	0.16	1.971	1.736
6	0.032	2.058	1.823
7	0.0064	1.92	1.685
8	0.00128	1.916	1.681
9	0.000256	1.915	1.68
10	0.0000512	1.916	1.681

Table S1. Raw data of TR-FRET assay for Compound 1a

Compound 1b

SL No.	1b Concentration (μM)	Raw Data (RLU)	Raw Data - Background (RLU)
1	100	0.421	0.268479758
2	20	1.199	1.025077942
3	4	1.779	1.596466248
4	0.8	2.012	1.7564370579
5	0.16	1.896	1.7237853418
6	0.032	1.859	1.568017542
7	0.0064	1.857	1.671486208
8	0.00128	1.865	1.732455786
9	0.000256	1.849	1.754676693
10	0.0000512	2.014	1.766631767

Table S2. Raw data of TR-FRET assay for Compound 1b

Compound 1c

SL No.	1c Concentration (μM)	Raw Data (RLU)	Raw Data - Background (RLU)
1	100	0.06866093	-0.146011744
2	20	0.161709165	0.02703648
3	4	1.330425732	1.095753048
4	0.8	1.99103139	1.756358706
5	0.16	1.994489916	1.759817232
6	0.032	1.901858191	1.657185506
7	0.0064	1.885644002	1.65097132
8	0.00128	1.880000000	1.637093772
9	0.000256	1.880000000	1.646174163
10	0.0000512	1.881399226	1.646726544

Table S3. Raw data of TR-FRET assay for Compound 1c

Compound 1d

SL No.	1d (Concentration (μM)	Raw Data (RLU)	Raw Data - Background (RLU)
1	100	0.652665928	0.4875893
2	20	0.592964879	0.49696625
3	4	1.542119287	1.307067659
4	0.8	2.017664099	1.890487471
5	0.16	1.945355712	1.780279084
6	0.032	1.990302541	1.875225913
7	0.0064	2.061302881	1.836226253
8	0.00128	2.000000000	1.790431041
9	0.000256	2.040000000	1.881119219
10	0.0000512	2.020297283	1.875698312

Table S4. Raw data of TR-FRET assay for Compound 1d

Compound 1e

SL No.	1d (Concentration (μM)	Raw Data (RLU)	Raw Data - Background (RLU)
1	100	0.834	0.586
2	20	1.018	0.721
3	4	1.752	1.522

4	0.8	1.820	1.683
5	0.16	1.922	1.688
6	0.032	1.988	1.752
7	0.0064	1.927	1.692
8	0.00128	1.903	1.678
9	0.000256	1.860	1.610
10	0.0000512	2.087	1.839

Table S5. Raw data of TR-FRET assay for Compound 1e

Compound 1f

SL No.	1d (Concentration (μM)	Raw Data (RLU)	Raw Data - Background (RLU)
1	100	0.7756898	0.942665928
2	20	0.79898825	0.862044774
3	4	1.247045656	1.542119287
4	0.8	1.88148727	2.055564099
5	0.16	1.711279181	1.945355712
6	0.032	1.766225913	1.990302541
7	0.0064	1.826226253	2.061302881
8	0.00128	1.764161041	2.000000000
9	0.000256	1.801119119	2.040000000
10	0.0000512	1.795220656	2.020297283

Table S6. Raw data of TR-FRET assay for Compound 1f
